# First report of *Blastocystis* sp. and *Enterocytozoon bieneusi* in raptors

**DOI:** 10.3389/fvets.2025.1538725

**Published:** 2025-03-19

**Authors:** Zhen-Qiu Gao, Si-Yuan Qin, Lin-Hong Xie, Guang-Rong Bao, Xingzhou Wang, Ya Qin, Xuetao Han, Xiaoming Yu, Cong-Cong Lei, Xiao-Tian Zhang, Ming-Yuan Yu, He-Ting Sun, Shuo Liu

**Affiliations:** ^1^School of Pharmacy, Yancheng Teachers University, Yancheng, Jiangsu, China; ^2^Center of Prevention and Control Biological Disaster, State Forestry and Grassland Administration, Shenyang, Liaoning, China; ^3^College of Veterinary Medicine, Qingdao Agricultural University, Qingdao, Shandong, China; ^4^Shandong Changdao National Nature Reserve Administration, Yantai, Shandong, China

**Keywords:** *Enterocytozoon bieneusi*, *Blastocystis* sp., raptor, prevalence, China

## Abstract

**Introduction:**

*Blastocystis sp*. and *Enterocytozoon bieneusi* are common zoonotic pathogens threatening human and animal health. These parasites are widely distributed in birds, and substantial research on their prevalence has been conducted. However, no studies on *Blastocystis* sp. and *E. bieneusi* in raptors exist.

**Methods:**

The present study collected 335 fecal samples from raptors in Changdao, China. The prevalence and genotypes of *Blastocystis* sp. and *E. bieneusi* were determined through amplification of *SSU* rRNA and ITS gene. Phylogenetic analysis was performed using MEGA 11 with the neighbor-joining method (Kimura 2-parameter model, 1000 replicate).

**Results:**

The overall infection rates of *Blastocystis* sp. and *E. bieneusi* in raptors were 1.19% (4/335) and 1.79% (6/335), respecttively. Among them, the highest infection rate of *Blastocystis* sp. was observed in *Accipiter nisus* (3.85%, 1/26), while *Buteo japonicus* showed the highest infection rate of *E. bieneusi* (33.33%, 1/3), followed by *Asio otus* (7.69%, 1/13). This study identified two *Blastocystis* sp. subtypes: ST3 and ST10 in raptors for the first time. Regarding *E. bieneusi* in raptors, we identified four genotypes: CHN-F1, HND-III, BEB6, and HLJD-I. Among these, BEB6 and CHN-F1 are notable for their zoonotic potential and the risk of waterborne outbreaks.

**Discussion:**

These findings suggest that raptors may be potential transmitters of *Blastocystis* sp. and *E. bieneusi* to humans and other animals, as well as sources of water contamination. This study fills a gap in the research on *Blastocystis* sp. and *E. bieneusi* in raptors and is important for public health safety.

## Introduction

*Blastocystis* sp. is a zoonotic intestinal protozoan of the family *Blastocystidae*, which can be transmitted through foodborne, waterborne, person-to-person, and zoonotic routes (1). Approximately one billion people worldwide are infected with this parasite, with infection rates among diarrhea patients ranging from 0.8 to 100% (2). However, a large number of *Blastocystis* sp. have been detected in both symptomatic and asymptomatic individuals (3). Therefore, their role in the host's gut (whether as a symbiont or a parasite) remains unclear, and their pathogenicity has yet to be conclusively determined. *Blastocystis* sp. exhibits significant genetic diversity, with 44 subtypes reported, of which 38 STs have been confirmed as valid subtypes. Studies show that 14 subtypes, including ST1–ST10, ST12, ST14, ST16, and ST23, are recognized as zoonotic subtypes (4). Approximately 90% of human infections are caused by ST1–ST4, with ST3 being the dominant subtype (5). Other subtypes are primarily found in other animals (6).

Microsporidia are a highly diverse group of parasites, both in terms of host range and genetic variation (7). Over 200 genera and more than 1,700 species are known globally, capable of infecting up to 240 species of vertebrates and invertebrates (8). Among human infections caused by microsporidia, *Enterocytozoon bieneusi* is the most common pathogenic species, accounting for approximately 90% of all microsporidia infections in humans (9). The pathogenicity of *E. bieneusi* is closely linked to the host's immune status (10). In individuals with normal immune function, the infection is typically asymptomatic or presents with mild symptoms. However, in immunocompromised individuals, such as those with HIV, the infection can lead to diarrhea, abdominal pain, and, in severe cases, even death (11). Based on analysis of the Internal Transcribed Spacer (ITS) nucleotide sequence, 820 genotypes of *E. bieneusi* have been identified and further categorized into 15 phylogenetic groups (12). Group 1 includes most zoonotic genotypes, which exhibit strong cross-species transmission capabilities and high zoonotic potential (12). Group 2 primarily infects ruminants, although some genotypes (such as BEB4, BEB6, I, and J) have been reported to infect humans. Groups 3–15 exhibit stronger host specificity, and their zoonotic potential is either limited or unclear (8).

As global ecosystems change, the habitats of wildlife are shrinking, especially under the intensified influence of human activities. As a result, the interaction space between domesticated and wild animals has become increasingly complex. Raptors, as apex predators in the food chain, have close ecological interactions with various poultry, livestock, and other wildlife, making them potential carriers for parasite transmission. Studies have shown that the prevalence of *Blastocystis* sp. in global bird populations is 29% (2). In addition, a meta-analysis indicates that the infection rate of microsporidia in birds worldwide is 14.6%, with *E. bieneusi* accounting for 77.42% of microsporidian infections (13). These findings suggest that both pathogens are widely distributed among birds. If raptors were infected with these two parasites, it would not only threaten their health but also, through ecological interactions, may impact other species and even human health. However, there have been no reports of raptors being infected with *Blastocystis* sp. and *E. bieneusi*. This knowledge gap hinders our understanding of the role of raptors in the transmission of these parasites and their potential impacts on human and animal health.

This study is the first to investigate the prevalence and genetic diversity of *Blastocystis* sp. and *E. bieneusi* in raptors. The findings will provide data to support ecological restoration and public health safety and offer valuable insights into the transmission risks of infectious diseases in raptors and potential preventive measures.

## Materials and methods

### Samples collection

From September to October 2024, a total of 335 samples were collected form raptors in Shandong Changdao National Nature Reserve, including *Caprimulgus jotaka* (*n* = 2), *Asio flammeus* (*n* = 3), *Accipiter gentilis* (*n* = 3), *Pernis ptilorhynchus* (*n* = 2), *Otus sunia* (*n* = 242), *Otus lettia* (*n* = 1), *Falco tinnunculus* (*n* = 2), *Buteo japonicus* (*n* = 3), *Accipiter nisus* (*n* = 26), *Accipiter gularis* (*n* = 28), *Accipiter virgatus* (*n* = 4), *Falco subbuteo* (*n* = 1), *Ninox scutulata* (*n* = 2), *Falco peregrinus* (*n* = 3), *Asio otus* (*n* = 13). We captured raptors using nets on Daheishan Island, Changdao, and released them after collecting cloacal swab samples. During sampling, we recorded the species and sampling time. All samples were transported to the laboratory on dry ice and stored at −80°C. All sampling procedures strictly adhered to the guidelines of the Ethics Committee of Qingdao Agricultural University.

### DNA extracting and PCR amplifying

After adding 500 μL of physiological saline to each cloacal swab, the mixture was vortexed at maximum speed until the feces were completely detached from the swab. According to the manufacturer's instructions, DNA was extracted using the E.Z.N.A. Stool DNA Extraction Kit (Omega Biotek Inc, Norcross, GA, USA). The extracted DNA samples were stored at −20°C until PCR analysis.

Nested PCR was performed to amplify the Internal Transcribed Spacer (ITS) region to detect the presence of *E. bieneusi* in the samples. In the first round, external primers NEBF1 (5′-GGTCATAGGGATGAAGAG-3′) and NEBR1 (5′-TTCGAGTTCTTTCGCGCTC-3′) were used, with the following reaction program: 94 for 5 min for pre-denaturation; 94°C for 45 s, 55°C for 45 s, 72°C for 1 min, for 35 cycles; and a final extension at 72°C for 10 min. In the second round, internal primers NEBF2 (5′-GCTCTGAATATCTATGGCT-3′) and NEBR2 (5′-ATCGCCGACGGATCCAAGTG-3′) were used, with an extension time of 40 s and other reaction conditions the same as the first round (14). Additionally, forward primer RD5 (5′-ATCTGGTTGATCCTGCCAGT-3′) and reverse primer BhRDr (5′-GAGCTTTTTAACTGCAACAACG-3′) were used to amplify the small subunit ribosomal RNA (*SSU* rRNA) gene to detect the presence of *Blastocystis* sp. in the samples, under the following conditions: 94°C for 5 min for pre-denaturation; 94°C for 45 s, 57°C for 45 s, 72°C for 1 min, for 35 cycles; and a final extension at 72°C for 10 min (15). All PCR reactions included positive and negative controls. PCR products were analyzed by electrophoresis on a 1.5% agarose gel to assess the amplification results.

### Sequencing analysis

All PCR-positive products were subjected to bidirectional sequencing by Qingdao Weilai Biotechnology Co., Ltd. Representative sequences were obtained by clustering at 100% similarity using cd-hit. These representative sequences were then compared with reference sequences from GenBank. A phylogenetic tree was constructed in MEGA 11 software using the neighbor-joining method (NJ) combined with the Kimura 2-parameter model, and the reliability of the results was assessed through 1,000 bootstrap replicates.

### Statistical analysis

Data analysis was performed using SPSS software (IBM Corp., Armonk, NY, USA), calculating the odds ratio (OR) and 95% confidence intervals (95% CI) of the infection rate of parasites. A *p*-value of < 0.05 was considered statistically significant.

## Results

### Prevalence of *Blastocystis* sp. and *E. bieneusi* in raptors

**Figure 1:**
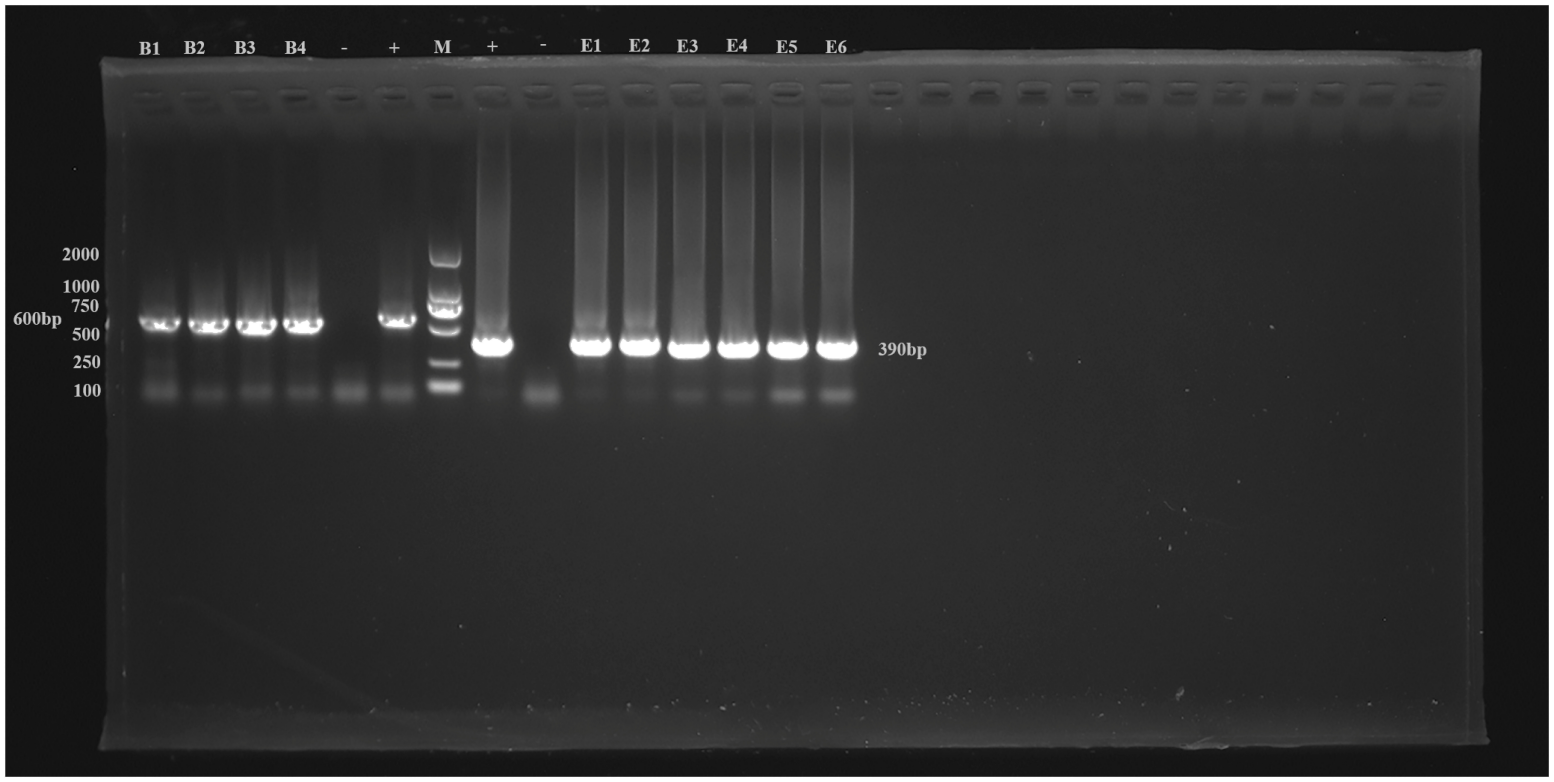
PCR amplification results for the *SSU* rRNA gene of *Blastocystis* sp. and the ITS gene of *E. bieneusi*. B1–B4 represent *Blastocystis* sp.-positive samples, E1–E6 represent *E. bieneusi-*positive samples. –, Negative control; +, Positive control; M, Marker.

In this study, four *Blastocystis* sp*.-*positive samples were detected from 335 raptor fecal swab samples, with an overall prevalence was 1.19% (4/335, 95% CI 0.33–3.03). No significant differences in prevalence were found between different raptor species (χ^2^ = 4.45, *df* = 14, *P* = 0.9921). Among the species, *Accipiter nisus* had the highest prevalence (3.85%, 1/26), followed by *Otus sunia* (1.24%, 3/242). No *Blastocystis* sp. infection was detected in other species (Table 1, Figure 1).

**Table 1:** Prevalence of *Blastocystis* sp. in raptors.

**Species**	**No. examined**	**No. positive**	**% (95% CI)**	**OR**	**Subtype (*n*)**
*Otus sunia*	242	3	1.24% (0.15–3.12)	Reference	ST10 (2) ST3 (1)
*Caprimulgus jotaka*	2	0	0 (-)	-	-
*Asio flammeus*	3	0	0 (-)	-	-
*Accipiter gentilis*	3	0	0 (-)	-	-
*Pernis ptilorhynchus*	2	0	0 (-)	-	-
*Otus lettia*	1	0	0 (-)	-	-
*Falco tinnunculus*	2	0	0 (-)	-	-
*Buteo japonicus*	3	0	0 (-)	-	-
*Accipiter nisus*	26	1	3.85% (0.00–15.77)	3.19 (0.32–31.80)	ST10 (1)
*Accipiter gularis*	28	0	0 (-)	-	-
*Accipiter virgatus*	4	0	0 (-)	-	-
*Falco subbuteo*	1	0	0 (-)	-	-
*Ninox scutulata*	2	0	0 (-)	-	-
*Falco peregrinus*	3	0	0 (-)	-	-
*Asio otus*	13	0	0 (-)	-	-
Total	335	4	1.19% (0.33–3.03)	-	-

CI, confidence interval.

Additionally, six positive samples of *E. bieneusi* were detected in this study, resulting in an overall infection rate of 1.79% (6/335, 95% CI 0.66–3.86). The highest infection rate was found in *Buteo japonicus* (33.33%, 1/3), followed by *Asio otus* (7.69%, 1/13), *Accipiter gularis* (3.57%, 1/28), and *Otus sunia* (1.24%, 3/242). No *E*. *bieneusi* infection was found in other species. The differences in infection rates between species were not statistically significant (χ^2^ = 9.45, *df* = 14, *P* = 0.8011; Table 2, Figure 1).

**Table 2:** Prevalence of *Enterocytozoon bienuesi* in raptors.

**Species**	**No. examined**	**No. positive**	**% (95% CI)**	**OR**	**Genotype (*n*)**
*Otus sunia*	242	3	1.24 (0.15–3.12)	Reference	HLJD-I (2), BEB6 (1)
*Caprimulgus jotaka*	2	0	0.00 (-)	-	-
*Asio flammeus*	3	0	0.00 (-)	-	-
*Accipiter gentilis*	3	0	0.00 (-)	-	-
*Pernis ptilorhynchus*	2	0	0.00 (-)	-	-
*Otus lettia*	1	0	0.00 (-)	-	-
*Falco tinnunculus*	2	0	0.00 (-)	-	-
*Buteo japonicus*	3	1	33.33 (0.00–94.11)	39.83 (2.80–567.67)	CHN-F1 (1)
*Accipiter nisus*	26	0	0.00 (-)	-	-
*Accipiter gularis*	28	1	3.57 (0.00–14.70)	2.95 (0.30–29.37)	HND-III (1)
*Accipiter virgatus*	4	0	0.00 (-)	-	-
*Falco subbuteo*	1	0	0.00 (-)	-	-
*Ninox scutulata*	2	0	0.00 (-)	-	-
*Falco peregrinus*	3	0	0.00 (-)	-	-
*Asio otus*	13	1	7.69 (-)	-	BEB6 (1)
Total	335	6	1.79 (-)	-	-

CI, confidence interval.

### Subtypes/genotypes distribution of *Blastocystis* sp. and *E. bieneusi* in raptors

Through sequence analysis of *Blastocystis* sp.-positive samples, this study identified two *Blastocystis* sp. subtypes, ST10 and ST3, with ST10 being the dominant subtype (75%, 3/4), found in both *Accipiter nisus* and *Otus sunia*. ST3 was detected only in *Otus sunia* (Table 1).

Additionally, sequence analysis of six *E. bieneusi-*positive samples identified four genotypes: HLJD-I, BEB6, HND-III, and CHN-F1. BEB6 (33.33%, 2/6) was found in both *Asio otus* and *Otus sunia*, with HLJD-I (66.66%, 2/3) being the dominant genotype in *Otus sunia*. HND-III and CHN-F1 were detected in *Accipiter gularis* and *Buteo japonicus*, respectively (Table 2).

### Phylogenetic relationship of *Blastocystis* sp. subtypes and *E. bieneusi* genotypes

**Figure 2:**
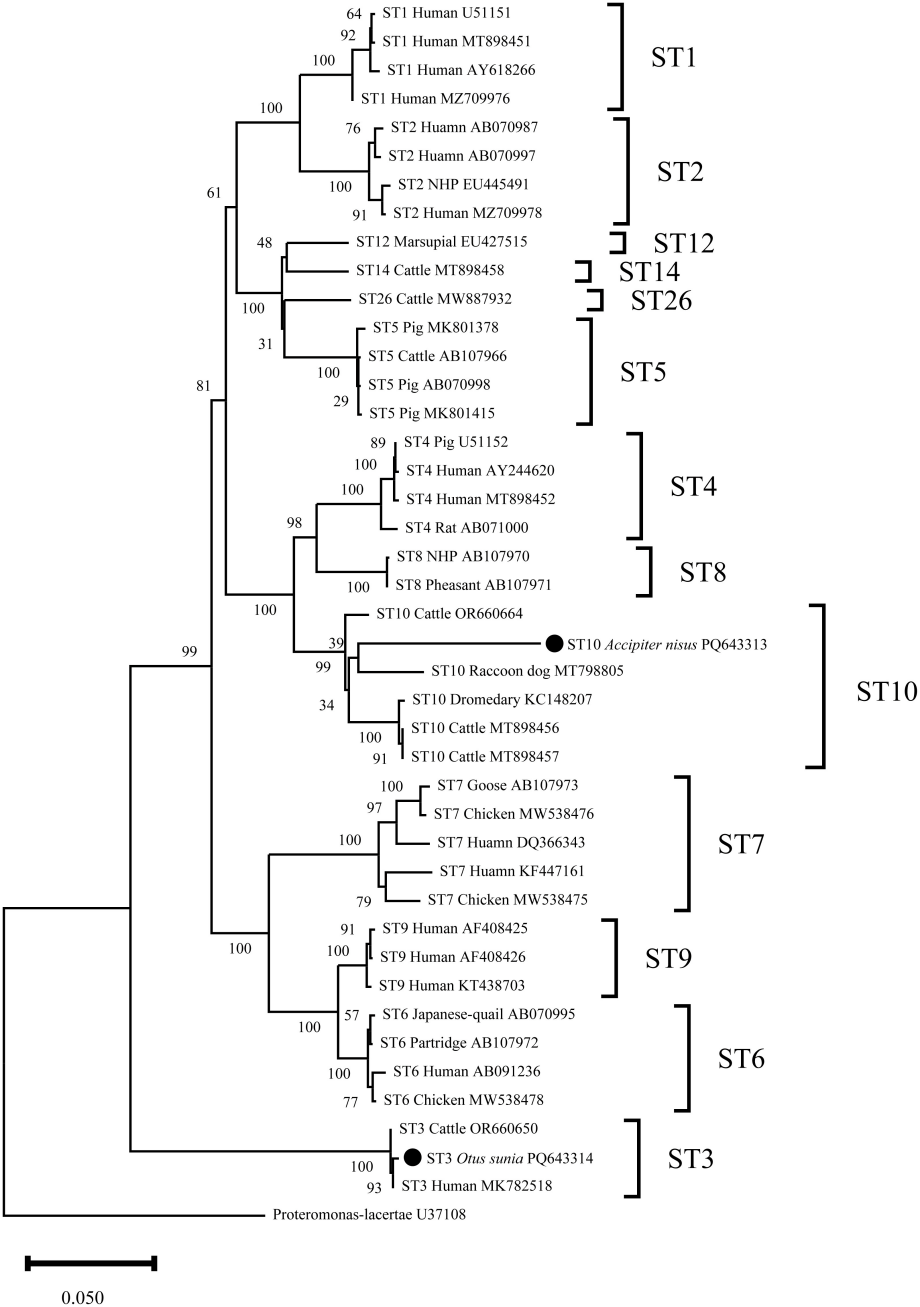
Phylogenetic relationship between the *Blastocystis* sp. sequences in this study and the reference sequences downloaded from GenBank was assessed using the neighbor-joining (NJ) method, with genetic distances calculated based on the Kimura 2-parameter model. The nucleotide sequences identified in this study are marked with black circles before the subtype names. The numbers on the branches represent the bootstrap percentage values from 1,000 repetitions, with values >50% shown in the tree.

This study obtained two representative sequences of *Blastocystis* sp. through clustering. Phylogenetic analysis showed that PQ643313 clustered with the reference sequence MT798805 in the same clade, both belonging to the ST10 subtype. PQ643314 showed 100% similarity with the human-derived reference sequence MK782518 and clustered in the ST3 subtype (Figure 2).

**Figure 3:**
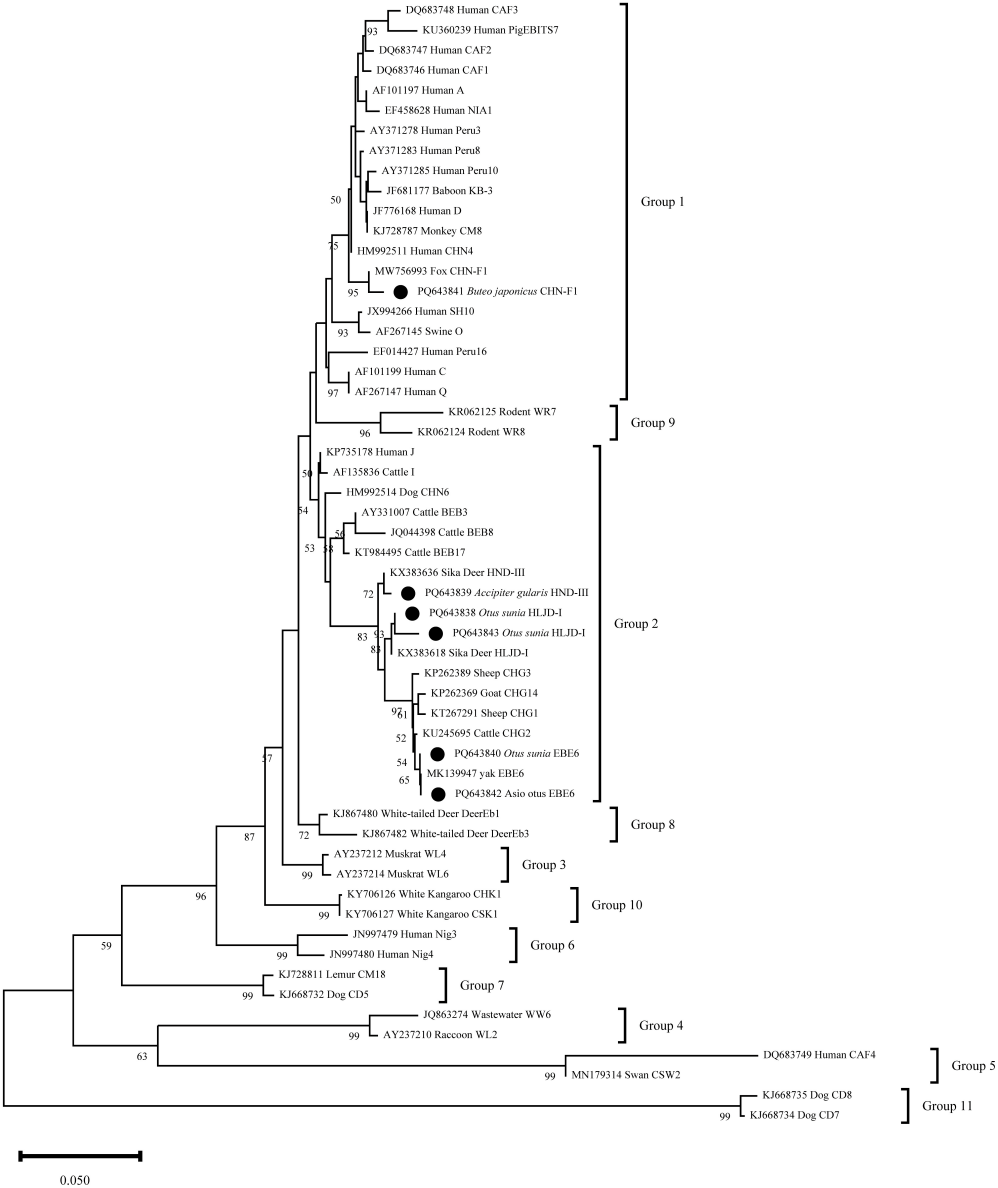
Phylogenetic relationship between the *E. bieneusi* sequences in this study and the reference sequences downloaded from GenBank was assessed using the neighbor-joining (NJ) method, with genetic distances calculated based on the Kimura 2-parameter model. The nucleotide sequences identified in this study are marked with black circles before the subtype names. The numbers on the branches represent the bootstrap percentage values from 1,000 repetitions, with values >50% shown in the tree.

The phylogenetic analysis of six representative *E. bieneusi* sequences revealed that PQ643841 shared 99% similarity with the reference sequence MW756993 and clustered in the first group. The other representative sequences clustered in the second group, with PQ643838 and PQ643843 located in the same branch. These two sequences, along with the reference sequence KX383618, formed a sister group and all belong to the HLJD-I genotype. PQ6463839 showed 99% similarity with the reference sequence KX383636 and formed a separate branch. PQ643840 and PQ643842 are of the BEB6 genotype and clustered with the reference sequence MK139947 in the same clade (Figure 3).

## Discussion

A meta-analysis has shown that the global prevalence of *Blastocystis* sp. in birds is 29% (2). The prevalence is 35% in Chinese peafowls, 17.8% in Malaysian quails, and 42.9% in Iranian pigeons (16–18). These data indicate that *Blastocystis* sp. is widely distributed among birds globally. In this study, the overall prevalence of *Blastocystis* sp. in raptors was 1.19%, which is lower than the 4.92% prevalence reported in migratory birds in Northeast China (19). This difference may be attributed to sampling methods, geographic location, and other factors, and the specific reasons warrant further investigation. Additionally, the highest prevalence was observed in *Accipiter nisus* (3.85%, 1/26), followed by *Otus sunia* (1.24%, 3/242), with no *Blastocystis* sp. infection detected in other raptor species. This variation could be linked to the host's immune status. As a common parasite in birds, *E. bieneusi* had an overall infection rate of 1.79% (6/335) in this study, which is lower than the infection rates in Central European pigeons (13.29%), Iraqi pet birds (32.32%), and Chinese swans [7.49%; (20–22)]. The low prevalence in raptors may indicate a relatively low exposure or low suitability of raptors as hosts of *E. bieneusi* compared to other birds. Furthermore, the infection rates of *E. bieneusi* in different raptor species ranged from 0 to 33.33%, with the highest infection rate observed in *Buteo japonicus* (33.33%). This difference may be related to the the sample size as well as the differences in susceptibility among different species to parasites.

The known *Blastocystis* sp. subtypes that can infect humans range from ST1 to ST10, with ST6 and ST7 being widespread in various bird species such as poultry, peafowls, and quails, and are considered the dominant subtypes in birds (16, 17, 23). However, the results of this study differ from other bird studies. We did not detect ST6 or ST7 in raptors, but instead unexpectedly identified ST3 and ST10. Epidemiological data show that ST3 is the primary subtype responsible for human infections and is widely found in dogs, rodents, and cattle, indicating that this subtype has strong host adaptability and significant zoonotic potential (24–26). ST10 is widely distributed in animals such as cattle, sheep, and camels, and is considered one of the most specific subtypes in ruminants (24, 27, 28). The infection of ST3 and ST10 in raptors is likely due to interactions with domesticated or wild animals and there is also a risk of cross-species transmission to humans, particularly among birdwatchers. Moreover, the increasing encroachment of human activities into wildlife habitats may facilitate such cross-species transmission, highlighting the need for greater attention to zoonotic risks in wildlife monitoring. An important observation is that Noradilah et al. isolated ST10 from river water in Malaysia, further suggesting that water sources could serve as a significant route for the transmission of *Blastocystis* sp. (29). This further emphasizes the environmental context in which *Blastocystis* sp. transmission may occur, where water contamination from wildlife could contribute to broader ecological and public health risks.

This study identified four genotypes of *E. bieneusi* from raptors: CHN-F1, HLJD-I, BEB6, and HND-III. CHN-F1 is classified into Group 1, initially discovered by Zhao et al. in foxes in China and widely present in farmed fur animals in China (30, 31). Subsequently, Holubová et al. reported this genotype in pigeons in Central Europe (18). Although no human infections have been reported so far, CHN-F1 has the potential to cause zoonotic diseases, and its cross-species transmission ability should not be overlooked. HLJD-I, BEB6, and HND-III are classified into Group 2, which was initially considered to be specific to ruminants (8). However, subsequent studies have shown that genotypes such as BEB4, BEB6, I, and J have been found in humans and other animals, indicating that this group poses a public health risk (32). HLJD-I and HND-III were first discovered in sika deer in Heilongjiang and Henan, China, respectively, and this study is the first to detect these two genotypes in birds, suggesting that these genotypes are no longer restricted to their original hosts and may have cross-species transmission potential (33). To our knowledge, BEB6 was first discovered and named in cattle, but later studies confirmed that this genotype is more common in sheep and has also been sporadically found in non-human primates, raccoon dogs, and other animals, suggesting that it has low host specificity and high adaptability (22, 31, 34–36). Notably, as reported by Ye *et al*., this genotype has already been detected in a wastewater treatment facility in Zhengzhou, China (37), suggesting that raptors may contaminate surface water sources through their feces, thereby posing a potential risk for waterborne outbreaks. This finding further highlights the potential role of raptors in environmental pollution, and future research should focus on the ecological risks of waterborne transmission of zoonotic pathogens.

However, this study has some limitations. For example, the sample size for certain raptor species is relatively small, which may affect the accuracy of infection rate estimates, especially for rare species. Additionally, the study was conducted in a single geographic location, which may not fully reflect the distribution patterns of these parasites in other areas. Another limitation is the lack of consideration of seasonal variation in parasite prevalence, which is an important factor in understanding the dynamics of infection. Future research should focus on expanding the sample size, especially for rare species, and incorporating multiple geographic regions to capture a broader range of ecological and environmental gradients. Additionally, implementing long-term monitoring to assess the seasonal and temporal trends of parasite prevalence will greatly enhance our understanding of the epidemiological patterns of raptor parasitic infections.

## Conclusion

In conclusion, this study is the first to reveal the prevalence of *Blastocystis* sp. and *E. bieneusi* in raptors, with infection rates of 1.19% and 1.79%, respectively. We identified two zoonotic *Blastocystis* sp. subtypes (ST3 and ST10), which were detected in birds for the first time. The study also identified four *E. bieneusi* genotypes (CHN-F1, HND-III, BEB6, and HLJD-I), among which BEB6 and CHN-F1, as zoonotic genotypes, were found in raptors for the first time. Both BEB6 and ST10 have the potential to cause waterborne outbreaks. These results suggest that raptors may be potential cross-species transmitters, creating new transmission pathways between humans and animals, and highlighting the potential ecological interactions and zoonotic risks between wild and domestic animals, particularly the threat of water contamination by raptors. Therefore, future research should focus on the interaction patterns between raptors, domestic animals, and other wildlife, and explore additional control strategies to reduce the risks of cross-species disease transmission and water contamination.

## Data Availability

The representative sequences of *Blastocystis* sp. obtained in this study have been submitted to GenBank, with sequence numbers PQ643313 and PQ643314. The representative sequences of *E. beneusi* have been submitted to GenBank, with sequence numbers: PQ643838-PQ643843.
